# Nonlinear Imaging Allows to Characterize Granulomas and Fibrosis in Mice Tissue From Experimental Schistosomiasis

**DOI:** 10.1002/jbio.70295

**Published:** 2026-06-04

**Authors:** Gladystone Rocha da Fonseca, Rennan R. M. Lima, Ronald Eduardo Soares, Bárbara Regina Melo Ribeiro, Paulo E. Cabral Filho, Adriana Fontes, Ana Maria de Paula

**Affiliations:** ^1^ Departamento de Física, Instituto de Ciências Exatas Universidade Federal de Minas Gerais Belo Horizonte Brazil; ^2^ Departamento de Biofísica e Radiobiologia Centro de Biociências, Universidade Federal de Pernambuco Recife Brazil

**Keywords:** collagen, fluorescence, nonlinear imaging, Schistosoma, SHG

## Abstract

Schistosomiasis is a neglected disease caused by parasites of the genus *Schistosoma* that poses serious public health problems. The liver is one of the organs seriously affected by the disease as it lodges *Schistosoma* eggs. We show results by nonlinear imaging techniques, Second Harmonic Generation and Two‐Photon Excitation Fluorescence, to characterize mice tissues infected with *Schistosoma mansoni* in an experimental model. We followed the evolution of the disease in liver tissue sections through three stages: 30, 60, and 120 days post‐infection—the pre‐patent, acute, and early chronic phases, respectively. The presence of the *Schistosoma* eggs causes an increase in the collagen content and also changes in its architecture. The results allow us to clearly evaluate the disease progress helping to improve the understanding of liver fibrosis.

Second Harmonic Generation and Two‐Photon Excitation Fluorescence microscopy allows to characterize mice tissues infected with *Schistosoma mansoni* in an experimental model.

## Graphical abstract

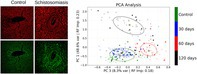

**Abbreviations**
SHG: second harmonic generation
TPEF: two‐photon excition fluorescence
WHO: World Health Organization

## 1 Introduction

The World Health Organization (WHO) has classified schistosomiasis as a tropical neglected disease and one of the most prevalent parasitosis affecting people worldwide. This designation has prompted the WHO to publish a road map that focuses on neglected diseases, highlighting the challenges related to schistosomiasis. The road map has encouraged the development of new diagnosis and therapeutic approaches; it has also stimulated the search for new tools to deepen the foundations of this disease and monitor treatment interventions [1, 2, 3]. Schistosomiasis is caused by helminths of the genus *Schistosoma*, and the main species capable of infecting humans are *S. haematobium*, *S. intercalatum* (with a great prevalence in Africa and the Middle East), *S. japonicum* (present especially in the Middle East), *S. mekongi* (prevalently in Southeast Asia), and *S. mansoni* (present in Africa, Caribbean, and South America). Humans are infected when cercariae of *Schistosoma* spp. penetrate their skin. Once inside the host, cercariae develop into schistosomula and migrate to key areas, commonly the hepatic portal and mesenteric veins, where they mature into adult worms. Afterward, male and female worms can generate at least 100 eggs per day, which are released into the water, allowing the entire parasite cycle to restart [1, 3, 4, 5]. During egg deposition in the organism, it can produce an inflammatory response in vital organs—especially the liver and kidney. First, liver damage exhibits a large volume of numerous primarily inflammatory cells (such as eosinophils and neutrophils) around the egg. Secondly, as the disease progresses, there is a replacement with newly formed connective tissue, with collagen deposition [6, 7].

Characterizing the inflammatory process and quantifying collagen deposition is important to verify the effectiveness of treatments [8]. Some studies have analyzed collagen deposition using tissue staining with Masson's trichrome [9, 10] and picrosirius red [10, 11, 12, 13, 14] under various biological conditions. Although both staining protocols have been widely used, especially in studies of fibrosis, they are laborious and limited in their ability to provide tissue structural analyses.

In this scenario, multiphoton microscopy by second harmonic generation (SHG) and two‐photon excitation fluorescence (TPEF) have become emergent methods for optical imaging with various applications in biological samples [15, 16, 17]. The SHG exhibits high responsiveness for non‐centrosymmetric structures, such as fibrillar collagens, and is sensitive to detailed quantification of collagen architecture [17, 18]. SHG, for example, has been applied to evaluate and investigate collagen deposition patterns in healthy and diseased tissues, being able to identify changes in the collagen type and organization without the need for exogenous labeling [18, 19, 20]. In addition, TPEF signals due to the tissue autofluorescence or specific staining dyes allow to complete the analysis of cellular regions of the tissue [19, 20]. Thus, SHG microscopy in conjunction with autofluorescence and computational analyses is a potential tool for examining collagen and inflammatory profiles in schistosomiasis.

Therefore, herein, we aimed to investigate collagen deposition patterns in liver tissue slices using SHG and TPEF microscopies, supported by computational analyses. Tissue samples from mice infected with *S. mansoni* were used as models. The experimental mouse model of schistosomiasis has proven to be appropriate for elucidating biological aspects across different disease stages, as it shares clinical and immunological similarities with humans [21, 22, 23, 24]. As far as our knowledge goes, this is a pioneering study applying SHG microscopy to schistosomiasis. We believe these evaluations could enhance our understanding of this parasitosis and contribute to the development of more effective diagnostic and treatment methods.

## 2 Materials and Methods

### 2.1 Tissue Samples

Slices were acquired from the Tissue Bank of the Pathology Sector of the Keizo Asami Immunopathology Laboratory, Federal University of Pernambuco, Brazil [Animal Research Ethics Committee (CEUA), process No. 23076.017619/2015‐60] [25]. Paraffinized liver tissue slices from mice infected with *S. mansoni* for intervals of 30, 60, and 120 days were obtained, representing nearly to the pre‐patent, acute, and early chronic phases of this parasitosis [26]. Controls were chosen to be liver tissue slices extracted from uninfected mice over the same time periods. The mice were infected with 50 cercariae to induce the disease, and subsequently euthanized for organ extraction. Tissue slices, 4 μm thick, were prepared using a microtome (CUT4062—Slee Mainz), and afterwards were fixed on slides [25]. Sections from different mice were cataloged according to disease stage (30, 60, and 120 days). We obtained images for four control mice and three mice for each disease stage (one liver section per mice). The number of regions of interest, acquired images per group, were 96, 67, 45 and 55 for the control, 30‐, 60‐, and 120‐days, respectively. For the control mice the tissue cuts showed no significative difference over the time periods, thus they are presented as one group.

### 2.2 Second Harmonic Generation and Two‐Photon Excitation Fluorescence Imaging

Before starting SHG and TPEF imaging, liver tissue slices non‐infected and infected with schistosomiasis of different stages (30, 60, and 120 days) were first analyzed by bright field optical microscopy. The aim was to capture wide and full image (in high‐definition) of each slide to identify areas containing granulomas. The images are presented in the Supporting Information, Figure S1 to S5. For the control and 30 days slices, that showed no granulomas, random regions were selected and also regions around the veins were imaged. We just visually avoided clear defected regions, giving SHG signals possibly from extraneous physiological collagen, as discussed in reference [15]. For the 60 and 120 days, we identified and imaged the regions containing granulomas.

The SHG and TPEF imaging were performed in a home‐modified confocal fluorescence microscope (Olympus FV300) using a Titanium‐Sapphire laser (Coherent Chameleon), operating at 800 nm, with a pulse width of 140 fs and a repetition rate of 80 MHz. The laser beam passed through the optics of the confocal fluorescence microscope scanning head and was focused on the sample using a 20× water immersion objective lens (numerical aperture, NA: 0.90). The average laser power was set to 5 mW at the sample. The backscattered SHG signal was collected by the same objective and directed to a photomultiplier (PMT) detector by a dichroic mirror (Semrock FF665‐Di02). A narrow bandpass filter (20 nm, Chroma HQ400/20 m‐2p) centered at the SHG wavelength (400 nm) and a blocking edge filter (Semrock FF01‐680/SP/25) were used to remove scattered laser light. For the TPEF microscopy, the dichroic mirror was removed from the beam path, enabling the fluorescence to be analyzed by the internal PMT of the confocal microscope. A bandpass filter (560–600 nm) and a blocking edge filter (Semrock FF01‐680/SP/25) were also applied in the TPEF system [19, 20]. This filter wavelength range covers the peak of the autofluorescence emission. The SHG and TPEF were acquired successively, at the same area of the tissue slices, in a scanning of 512 × 512 pixels (0.471 mm × 0.471 mm) with scan time of 2.71 s. In the backscattering geometry used in this work, the SHG signal is mainly from the fibrillar collagens [18].

The laser transmission through the sample is also acquired as an image in a PMT positioned after the microscope condenser. The transmission image can be collected simultaneously either with the SHG or the TPEF image. It was saved simultaneously with the TPEF image in a multi‐tiff image format. The images were collected at three different focal planes, separated by 1 μm. The acquisition of the three images allowed to assure the best focal position and also they were compared by the image analysis software to eliminate noise. Thus, the image acquisition time is 8.13 s for each SHG and TPEF + transmission images. For the tissue regions of interest with weak signals, the final images were obtained by accumulating about 3 scans for the TPEF images and 6 scans for the SHG images (the final image is the added up accumulations). However, the overall image acquisition time (SHG + TPEF + transmission) for each sample region of interest was at maximum around 1 min.

### 2.3 Image and Data Analysis

The images were analyzed by a Python software [19, 20] developed to extract numerical parameters from the SHG and TPEF signal (the software is available on GitHub [27]). The software details are described by Gomes et al. [20]. It is a general software that extract numerical features by analyzing the image intensity texture and fibrillar network. In brief, the intensity maps for the SHG, TPEF and transmission images are merged into a RGB image that allows to identify the fibrillar collagens and cellular regions (segment the image) and to extract quantitative parameters, in a total of 29 parameters as presented in Table S1. From the composed RGB image the fibrillar networks are identified in the image and the individual fibers are also identified [20]. Each separated fiber network is selected as a fiber segment (i.e., the number of fiber segments) and the cellular regions enclosed within the fibers network are identified as cellular segments (number of cell segments), see Table S1. Then, the parameters are calculated after the images are segmented into cellular and fibrillar regions, thus the parameter labels include the names “Cell Segment” or “Fiber Segment”, that are metrics obtained from the cellular regions or fibrillar regions in the image, respectively. The extracted parameters include direct intensity related metrics, as SHG and TPEF mean intensities, that are the average intensities for the image pixels in each segment. There are also intensity metrics that measure the intensity texture such as intensity standard deviation (STD) and intensity Shannon entropy, and the intensity angular distribution (Angle SDI). Shape parameters such as the segment circularity and eccentricity are also measured. The other parameters are traditional network metrics extracted for the fibrillar segments: network degree (average number of edges in each node of the network), network eigenvalue (network adjacency matrix maximum eigenvalue), network connectivity (connectivity of each node in the network) and network cross link density (average number of cross links (intersection of edges) per extracted fiber in the network), and the individual fiber number and the fibers average waviness (mean waviness (length over displacement) of the extracted fiber).

Before using the PyFiber software, we used the Otsu's threshold methodology to filter some autofluorescence signal from the SHG images. The TPEF signal is from the liver tissue autofluorescence, that shows a broadband intensity over the visible range, peaking around 580 nm. The SHG signal is mainly from the fibrillar collagens that are non‐centrosymmetric. However, as the autofluorescence is quite broad, there is some tail of TPEF signal also at the 400 nm that overlays with the SHG signal. This tail signal is more evident for the control and the 30 days images. Thus, to filter this autofluorescence signal from these images, we used the Otsu's threshold methodology [28, 29]. The Otsu's threshold method selects the threshold by maximizing the between‐class variance (or, conversely, minimizes the within‐class variance) of the two groups of pixels, here the autofluorescence and the SHG signal pixels. For the pixels identified as autofluorescence, we had the intensity reduced to one tenth of the original value.

The parameters were first tested for normality by the Shapiro–Wilk test and the homogeneity of variance test, Levene's test. For the parameters that passed normality, the statistical models Student's *t*‐test (for two groups) or ANOVA (for more than two groups) were performed. Otherwise, if one of the tests yields a p<0.05, the Mann–Whitney *U* test (for two groups) and the Kruskal–Wallis test (for more than two groups) were used. The performances of the ANOVA and Kruskal–Wallis tests were subsequently followed by post hoc tests when p‐values were below 0.05.

The 29 parameters are used to classify and automatically separate the tissue images from the control and different disease phases by unsupervised machine learning algorithms. We used Python standard scikit‐learn modules [29, 30]. The parameters were used as input for the Random Forest classifier to select the features. To identify the most relevant parameters and determine the optimal model architecture without optimization bias, we employed a Nested Cross‐Validation (Nested CV) approach. Within this framework, Recursive Feature Elimination with Cross‐Validation (RFECV) was coupled with a Random Forest estimator to recursively discard non‐informative features. Simultaneously, the inner loop of the Nested CV determined the optimal hyperparameters for the final Random Forest classifier to yield the best classification performance. The relative importance of the selected features is presented in Figure S6. In a parallel analysis to visually display the spatial separation among the experimental groups, Principal Component Analysis (PCA) was applied to reduce the data dimensionality. Instead of merely selecting the Principal Components (PCs) with the highest global variance, a Random Forest classifier was trained strictly on the PCA‐transformed training data. We then extracted the feature importance of the resulting PCs, and the two PCs exhibiting the highest importance for the Random Forest (representing the highest discriminative power) were selected to project the data into a 2D spatial map.

Lastly, we performed a robustness analysis to verify the reliability of these final metrics. For this purpose, we used four distinct random seeds (0, 4, 42, and 200) in the Repeated Stratified K‐Fold cross‐validation procedure, assessing the model's stability against data‐splitting randomness. For each random seed, the algorithm computed the class‐wise metrics. To visualize this variability, we plotted the mean Receiver Operating Characteristic (ROC), with the standard deviation represented by a shaded region. The Area Under the ROC Curve (AUC‐ROC) was also calculated as mean values across the four random seeds to attest to the model's consistent performance. The exceptional stability of the model against these variations is visually attested by the narrow confidence intervals (shaded standard deviations) around the ROC curves. Thus, the average performance consolidated by Nested CV statistically proves that the machine learning methodology outlined can abstract valid, robust, and reproducible biological patterns, regardless of which biological samples are randomly selected to compose the initial training stage.

## 3 Results

**FIGURE 1:**
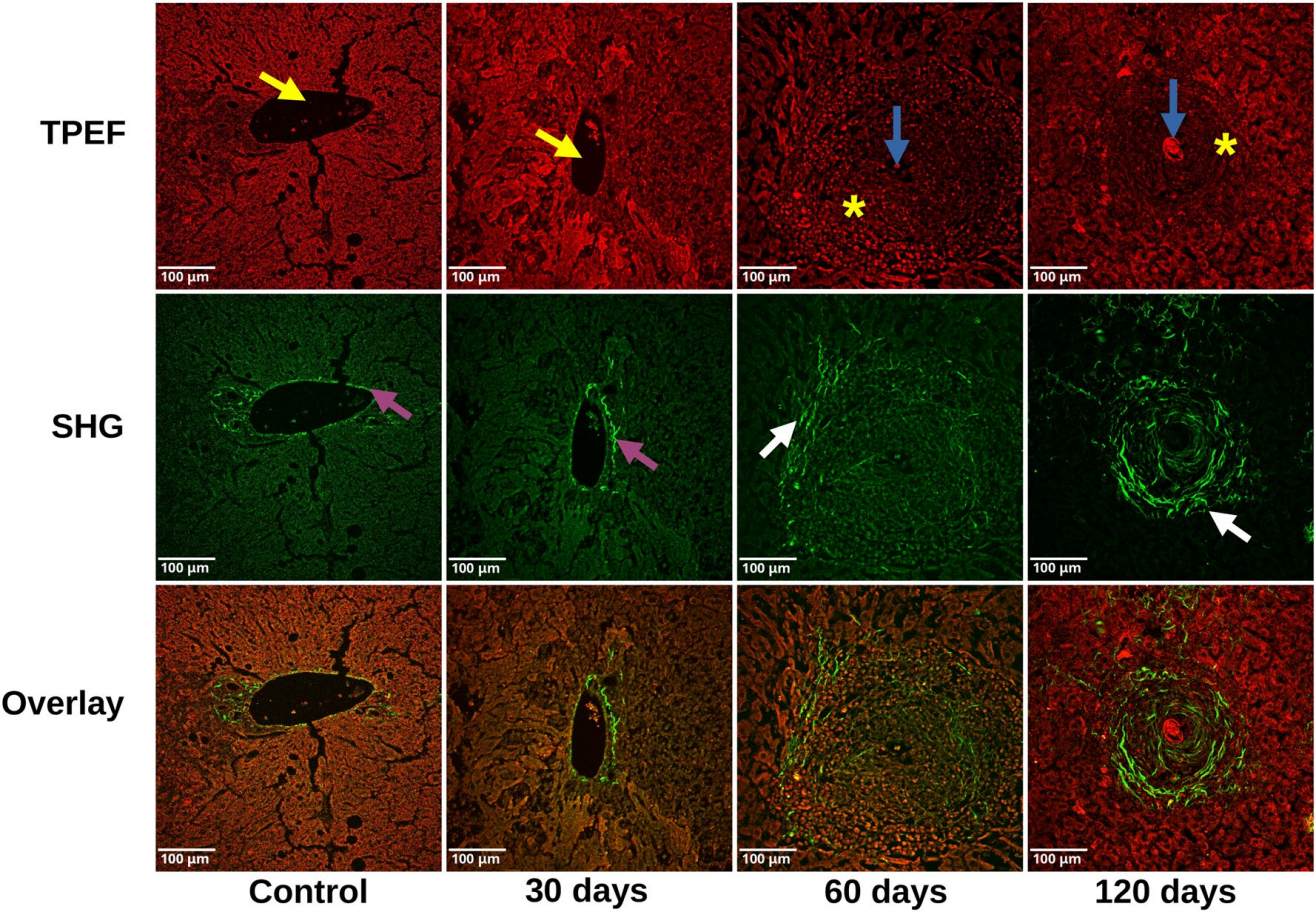
Representative images of the tissue cuts for control mice and mice after 30, 60, and 120 days of infection. Red images represent the TPEF signal from the tissue autofluorescence, green images the SHG signal from the fibrillar collagens and the bottom row displays overlays of the green and red channels as false‐color RGB maps to highlight the co‐localization of cells and fibers within the granulomas. The vein location in the control and 30‐days samples are indicated by yellow arrows in the TPEF images and the SHG signal around the veins is indicated by purple arrows. At the 60 and 120‐days images the parasite eggs are marked by blue arrows, the collagen fibers are indicated by white arrows. The small round granular spots at the TPEF images, marked by the yellow stars are showing the spots indicating shapes of possible inflammatory cells.

Figure 1 shows representative images of liver tissue cuts for the control mice and mice at the three disease stages, pre‐patent (30 days), acute (60 days) and early chronic (120 days) phases of infection. Red images correspond to the TPEF signal from liver tissue autofluorescence, while green images correspond to the SHG signal, mainly from the fibrillar collagens. The bottom row displays overlays of the green and red channels as false‐color RGB maps to highlight the co‐localization of cells and fibers within the granulomas. The control and 30‐days tissues present similar images. The 30‐days tissues show no eggs. For these images there is only a minor amount of collagen (indicated by purple arrows) around the veins (indicated by yellow arrows). However, the overall liver cells present a broadband autofluorescence with some signal also at the SHG image. In the acute phase (60 days), the granuloma around the egg (blue arrow) is mainly dominated by small round dots that are an indication of shapes of inflammatory cells (dots marked by a yellow star) and presents some round collagen fibers (white arrow). As discussed in the literature, these bulky granulomas are rich in inflammatory cells as a result of the host's immune response to the parasite's eggs [8, 31]. Also discussed in the literature, as the disease progresses to the chronic phase, the granulomas tend to reduce their sizes and become more fibrotic, thus at the early chronic phase the granuloma is dominated by circular thick collagen fibers around the eggs [8, 31]. That is also what is observed in the SHG image, circular fibers marked by the white arrow.

**FIGURE 2:**
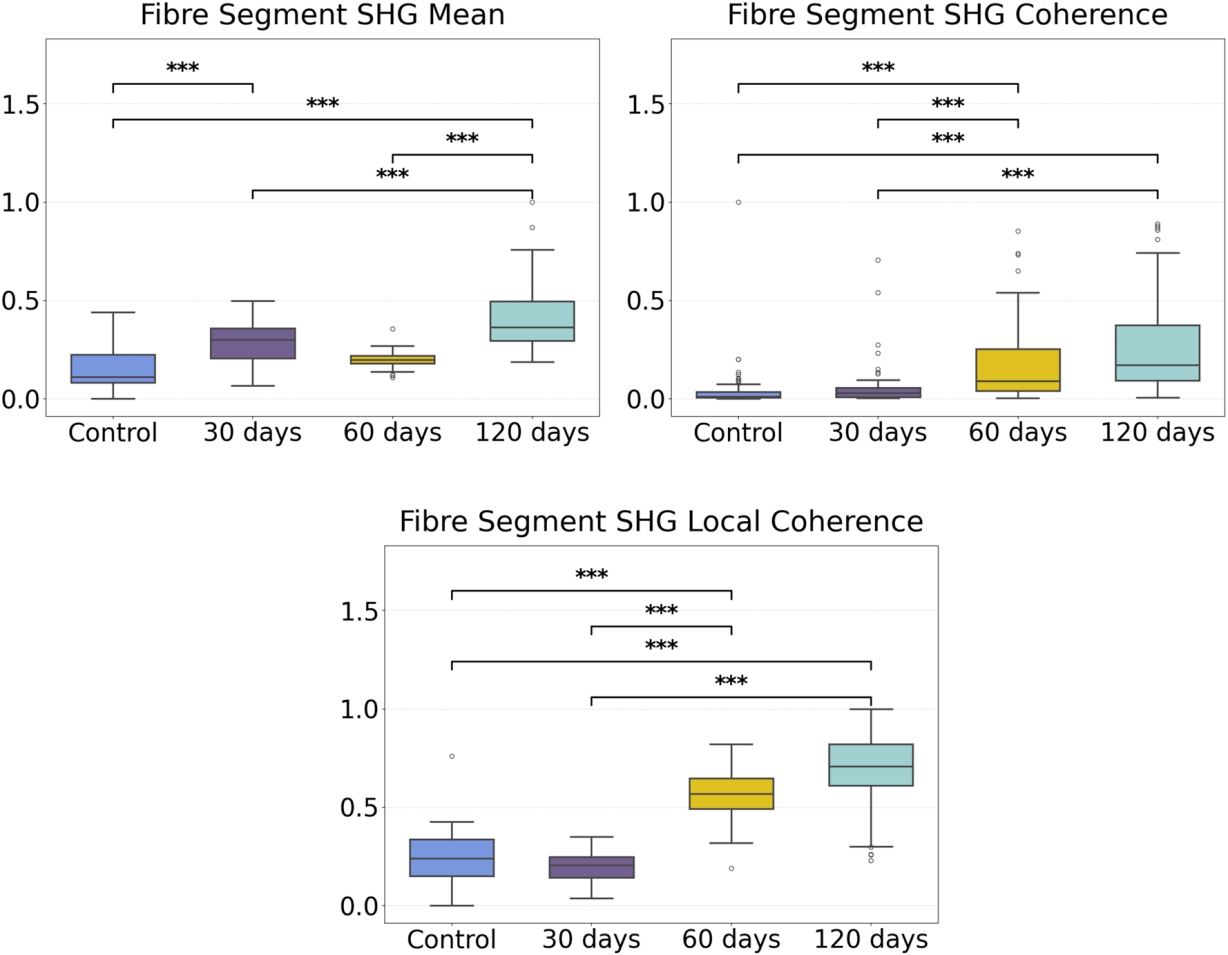
Boxplot graphics of the SHG mean intensity and collagen fibers coherence (degree of alignment of the collagen fibers) for all images of the 30‐, 60‐, and 120‐day tissues. The number of images are 96, 67, 45 and 55 for the control, 30‐, 60‐, and 120‐days liver sections, respectively. Significance between the groups is indicated by: *** for p<0.001.

By quantifying the SHG and TPEF intensities and architecture we obtain parameters to measure the fibrosis evolution as the disease progresses (parameters presented in the image and data analysis section). In Figure 2 we present parameters from the SHG signal that are more related to the observed round collagen fibers in the 60‐ and 120‐days images. The boxplots of all the parameters are presented in Figures S7 and S8. Figure 2 shows boxplot graphics of the SHG mean intensity (rescaled from 0 to 1) and the coherence (degree of alignment of the collagen fibers) for all the images of the control and 30‐, 60‐, and 120‐day tissues. The SHG mean intensity shows a large dispersion for the control and 30‐days images, but for the 60‐ and 120‐days images it increases as the disease progresses, highlighting the increase of the liver fibrosis. The coherence is a parameter that ranges from 0 to 1, where 0 means completely random fiber alignment and 1 means completely aligned fibers at one specific angle. The local coherence is the same parameter but measured locally in the image (3×3 pixels) and the coherence is the average alignment for the whole image area. As the granulomas are quite circular the overall image average fiber coherence is low, however the local coherence is high, as along the circular shape the fibers are well aligned. The coherence (fiber alignment) increases as the disease progresses. The Student's *t*‐test statistical significance among the groups is indicated by the stars. The mean SHG value and both local and global coherence allow good separation between most of the groups. The overall parameter boxplots are presented in the Supporting Information, Figures S7 and S8.

**FIGURE 3:**
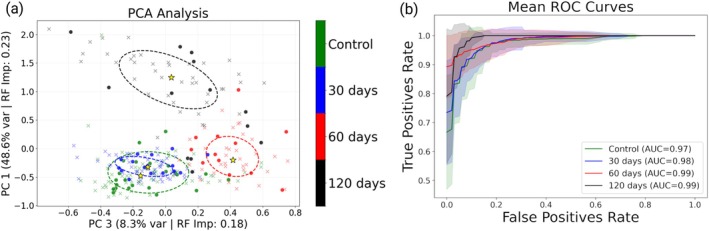
(a) PCA graphic along the two most relevant principal components, showing the separation between the tissue groups. The control and three disease stage groups are indicated by green (control), blue (30‐day), red (60‐day), and black (120‐day) symbols. The crosses represent the training images (75% of the images) and solid symbols the test images (25% of the images) for each group. (b) ROC curves for the separation between the tissue groups. The curves show the mean and the shades indicate the standard deviation obtained considering the different random seeds of the Random Forest classification.

Considering the overall parameters extracted from the images (Table S1) that evaluate the organization of the cells and fibers in the tissues, we use classification and machine learning techniques to separate the tissues according to the disease evolution. Figure 3a shows a 2D PCA projection considering all the 29 parameters (Table S1). The axes are the two PCs exhibiting the highest importance for the Random Forest, representing the highest discriminative power. The control and 30‐days groups are not separated, but they are separated from the other two disease stage groups. The stars represent the median of each group, and the ellipses enclose the data within one standard deviation. The crosses represent the training images (75% of the images) and solid symbols the test images (25% of the images) for each group. To evaluate the group separation we calculated the AUC‐ROC using selected parameters by the Random Forest as described in the Methods section. The performance of the machine learning classification is presented by the ROC curve in Figure 3b. The obtained AUC‐ROC values are all above 0.95, indicating an excellent classification performance.

## 4 Discussion

Fibrosis is related to many hepatic diseases [10]. Thus, characterizing the inflammatory process and quantifying collagen deposition in various organs is crucial for monitoring disease progression. Techniques that allow access and especially to quantify collagen deposition and structure organization are of major importance. The most used methods to study fibrosis are tissue staining with picrosirius red [10, 11, 13, 14] and Masson's trichrome [9, 10]. However, these methodologies are time‐consuming and dependent on the staining protocols. SHG imaging provides a more direct measurement of fibrillar collagen fiber and organization, with the advantage of allowing measurements in vivo or in fresh tissue sections, with no need for tissue processing [32].

A previous study based on picrosirius red staining showed that the young granulomas present thin collagen fibers, whereas the older granulomas, at the early chronic disease phase, are formed of thicker collagen fibers [12]. Another study, also based on picrosirius red staining, showed the presence of compact, concentric, and more organized collagen fibers, especially in the early chronic phase [33]. Our results corroborate these findings; the measured values of the mean SHG intensities in Figure 2 show the increase in collagen content as the disease progressed. In addition, we obtained the details of the collagen hierarchical organization, showing that the collagen fibers get more circular around the older granulomas. In addition, the 29 features extracted from the SHG and TPEF signal allowed us to monitor the changes around the egg at the granuloma formation in the acute disease phase (60 days) and the chronic phase (120 days).

The overall image parameters allowed us to quantify many of these granulomas' details, including the features that access also the shape of the inflammatory cells' presence, small round spots at the TPEF signal. The PCA tissue classification based on the extracted parameters, in Figure 3, clearly identifies the three disease stage groups (30, 60 and 120 days) separation. The control and 30 days groups are overlapped, indicating the early days of the disease cannot be separated by this image analysis. The accuracy and AUC‐ROC, with values close to 100%, present an excellent tissue classification. The features obtained from the TPEF and SHG image intensities, that measure the intensity texture, such as the pixel intensity angle distribution, entropy and fiber alignment (coherence and local coherence) access the organization of the cells and fibers in the granulomas. The results clearly indicate that the measured features provide quantitative details of the granuloma formation as described in the literature—the initial recruitment of cells around the entrapped egg, inducing an inflammatory response, followed by the collagen deposition [24].

Inflammatory and fibrosis effects were also observed in the heart tissue of mice in acute and chronic experimental models of murine schistosomiasis [34]. Thus, methodologies that can classify in detail the different stages of this disease would be an important tool to access and study the performance of new treatment drugs. Future works may include thicker tissue cuts or fresh tissue to try to obtain the three‐dimensional details of the fibrosis.

## 5 Conclusion

In conclusion, we obtained TPEF and SHG images that allowed us to follow the evolution of schistosomiasis, clearly showing the changes in liver tissue sections through the pre‐patent, acute, and early chronic phases. The presence of the *Schistosoma* eggs was observed for the acute (60 days) and early chronic (120 days) phases. At the acute phase there are hints of inflammatory cell shapes indicated by the spots in the TPEF images and at the chronic phase the collagen content increases, and also changes in its architecture is shown by the SHG images. The overall parameters obtained from the image analyses allowed to classify the disease stages with an accuracy of 0.886±0.026 and average AUC‐ROC of 0.982±0.010. Analyzing granuloma and fibrosis details is important in a range of disease control situations, for instance, it is essential in monitoring the effectiveness of new treatments. We showed results that allow to clearly evaluate the disease progress, improving the understanding of fibrosis and bringing future perspectives to this parasitosis.

## Author Contributions

Gladystone Rocha da Fonseca, Ronald Eduardo Soares, and Rennan R.M. Lima performed the experiments and data analyses. Gladystone Rocha da Fonseca, Ronald Eduardo Soares, Bárbara Regina Melo Ribeiro, and Ana Maria de Paula performed data analyses. Paulo E. Cabral Filho and Adriana Fontes provided the tissue samples. Ana Maria de Paula designed the nonlinear experiments. Ana Maria de Paula, Adriana Fontes, and Paulo E. Cabral Filho participated in the conceptualization of the study. All authors discussed the results and contributed to the manuscript writing.

## Funding

This work was supported by Conselho Nacional de Desenvolvimento Científico e Tecnológico. Fundação de Amparo à Pesquisa do Estado de Minas Gerais. Coordenação de Aperfeiçoamento de Pessoal de Nível Superior. Fundação de Amparo à Ciência e Tecnologia do Estado de Pernambuco.

## Disclosure

The authors have nothing to report.

## Conflicts of Interest

The authors declare no conflicts of interest.

## Supporting information


**Table S1:** Extracted metrics and their descriptions.
**Figure S1:** Bright field images of the tissue cuts for the control, 30, 60 and 120 days. The areas containing granulomas are indicated by the black arrows. The regions indicated by the red squares are presented in higher resolution in Figure S2 to S5, together with corresponding SHG and TPEF images of the marked regions of interest.
**Figure S2:** Control: the regions indicated by the red squares in Figure S1 are presented together with the Corresponding SHG and TPEF images.
**Figure S3:** 30 Days: the regions indicated by the red squares in Figure S1 are presented together with the Corresponding SHG and TPEF images.
**Figure S4:** 60 Days: the regions indicated by the red squares in Figure S1 are presented together with the Corresponding SHG and TPEF images.
**Figure S5:** 120 Days: the regions indicated by the red squares in Figure S1 are presented together with the Corresponding SHG and TPEF images.
**Figure S6:** Mean relative importance of the selected features by the Random Forest classifier algorithm.
**Figure S7 and S8:** Boxplots for the parameters extracted by the PyFibre software.

## Data Availability

The data that support the findings of this study are available from the corresponding author upon reasonable request.
