# Supplementary material for: Nonlinear Imaging Allows to Characterize Granulomas and Fibrosis in Mice Tissue From Experimental Schistosomiasis
Source: J Biophotonics. 2026 Jun 4;19(6):e70295. doi: 10.1002/jbio.70295 (PMC13238322; doi:10.1002/jbio.70295)
Supplement: Supplementary file 1 — Table S1: Extracted metrics and their descriptions. Figure S1: Bright field images of the tissue cuts for the control, 30, 60 and 120 days. The areas containing granulomas are indicated by the black arrows. The regions indicated by the red squares are presented in higher resolution in Figure S2 to S5, together with corresponding SHG and TPEF images of the marked regions of interest. Figure S2: Control: the regions indicated by the red squares in Figure S1 are presented together with the Corresponding SHG and TPEF images. Figure S3: 30 Days: the regions indicated by the red squares in Figure S1 are presented together with the Corresponding SHG and TPEF images. Figure S4: 60 Days: the regions indicated by the red squares in Figure S1 are presented together with the Corresponding SHG and TPEF images. Figure S5: 120 Days: the regions indicated by the red squares in Figure S1 are presented together with the Corresponding SHG and TPEF images. Figure S6: Mean relative importance of the selected features by the Random Forest classifier algorithm. Figure S7 and S8: Boxplots for the parameters extracted by the PyFibre software. [file JBIO-19-e70295-s001.pdf]

Nonlinear imaging allows to characterize granulomas and fibrosis in mice tissue from experimental Schistosomiasis

Gladystone Rocha da Fonseca[1], Rennan R. M. Lima[2], Ronald Eduardo Soares[1], Bárbara Regina Melo Ribeiro[1], Paulo E. Cabral Filho[2], Adriana Fontes[2], Ana Maria de Paula[1]

1 Departamento de Física, Instituto de Ciências Exatas, Universidade Federal de Minas Gerais, Belo Horizonte-MG, Brazil

2 Departamento de Biofísica e Radiobiologia, Centro de Biociências, Universidade Federal de Pernambuco, Recife-PE, Brazil

Journal of Biophotonics

Supporting information

Extracted Parameters

The quantitative parameters extracted from the images are obtained after segmenting the image into the cellular segment, that is provided by the TPEF image, and the fibre segment that are provided by the SHG image. The details of these extracted metrics are described by Gomes *et al.* [Journal of Biophotonics. 2023;16(6):e202200382. doi: https://doi.org/10.1002/jbio.202200382.]

They are listed in the following table.

Table S1
Extracted metrics and their descriptions.

Name	Description	Group
No. Segments	Number of segments in image.	Global
No. Fibres	Number of fibres extracted in image.	Global
Area	Area of segments in pix^2.	Segment
Coverage	Ratio of pixel area covered by segments to total area of image.	Segment
Intensity Mean	Mean intensity of all image pixels in segments.	Segment
Intensity STD	Pixel intensity standard deviation of all pixels in segments.	Segment
Intensity Entropy	Pixel intensities average Shannon entropy of all pixels in segments.	Segment
Angle SDI	Ratio of the mean to max values in the pixel angle distribution.	Segment
Coherence	Coherence of structure tensor constructed from all pixels in the segment.	Segment
Local Coherence	Average coherence of structure tensors for each pixel in the segment.	Segment
Circularity	The circumference of a circle with area equals to the segment area divided by the perimeter of the segment	Segment
Eccentricity	The focal distance of an ellipsoid with the same second-moments as the segment divided by its major axis length	Segment
Fibre Length	Mean length of the extracted fibres.	Fibre
Fibre Waviness	Mean waviness (length over displacement) of the extracted fibre.	Fibre
Fibre Network Degree	Average number of edges in each node of the reduced FIRE network.	Network
Fibre Network Eigenvalue	Reduced network adjacency matrix maximum eigenvalue.	Network
Fibre Network Connectivity	Connectivity of the reduced network	Network
Fibre Network Cross-Link Density	Average number of cross links (intersection of edges) per extracted fibre in the network	Network

Bright field images

The liver tissue sections were analyzed by bright field optical microscopy at the facilities of the LCPnano Laboratory (UFMG). The images were acquired using a CCD camera (DS-Ri2, Nikon) coupled to an optical microscope (Nikon ECLIPSE LV100ND), operating in episcopic configuration, with illumination provided by a 50 W halogen lamp. A 10× objective lens (Nikon TU Plan Fluor, NA 0.30) was used for the control, 60- and 120-days sections and a 20× (Nikon TU Plan ELWD, NA 0.40) for the 30-days section. Scan and acquisition control were performed using Nikon NIS-Elements software.

An automatic scanning mode was used for the acquisition of multiple frames, with a 10% overlap between adjacent images (without overlap for the 30-days section). The images of the whole tissue sections as shown in Figure S1 for the 30- , 60- and 120-day slides were composed using the image-stitching tool of the software. Small manual focus adjustments were performed every five frames. The exposure was automatically adjusted for each slide prior to the start of the scan and kept constant throughout the entire acquisition. The regions highlighted by the red squares are presented in Figures S2 to S5, together with the corresponding SHG and TPEF images, for the for the tissue cuts of control, 30, 60 and 120 days, respectively.

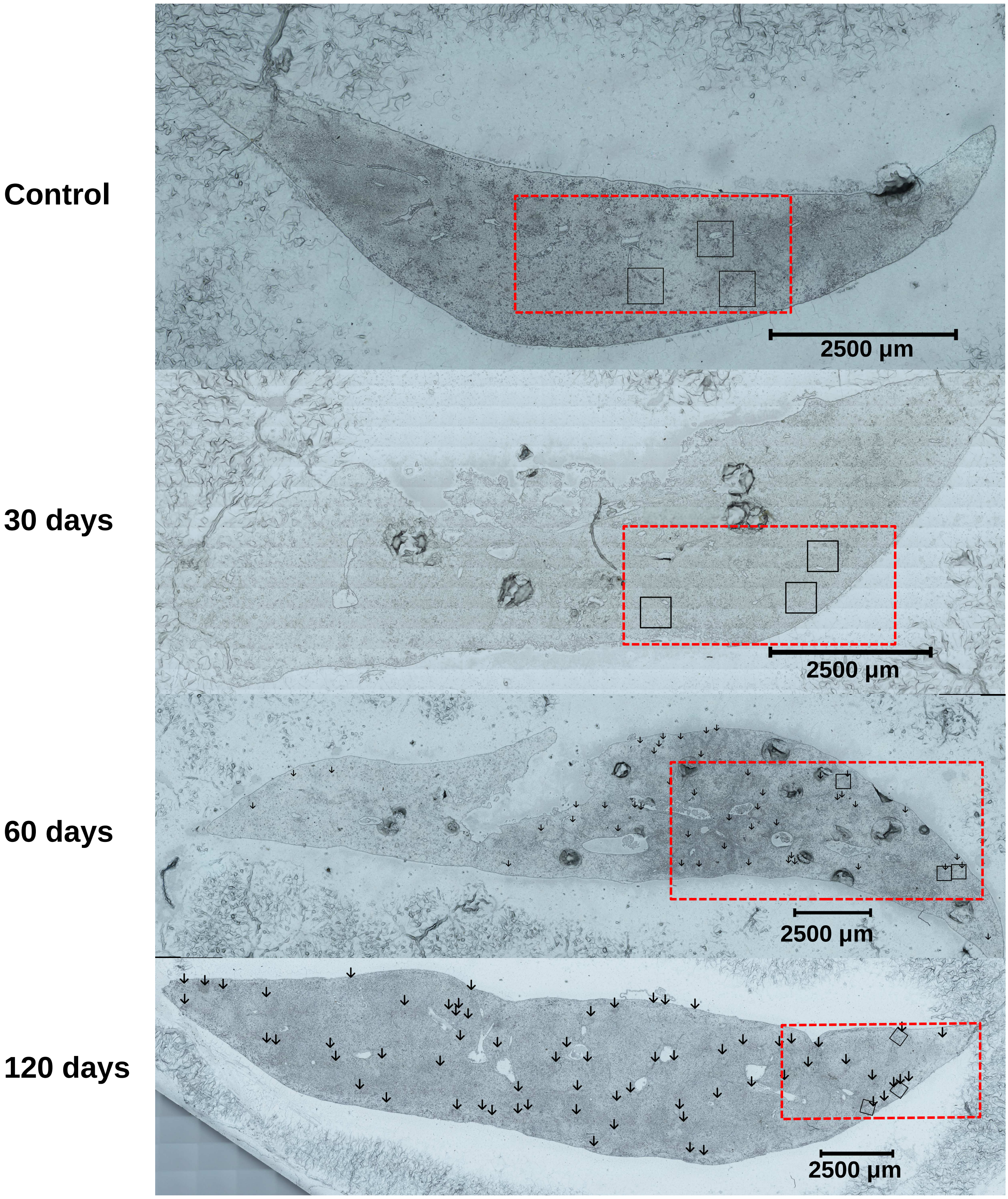

Figure S1
Bright field images of the tissue cuts for the control, 30, 60 and 120 days. The areas containing granulomas are indicated by the black arrows. The regions indicated by the red squares are presented in higher resolution in Figure S2 to S5, together with corresponding SHG and TPEF images of the marked regions of interest.

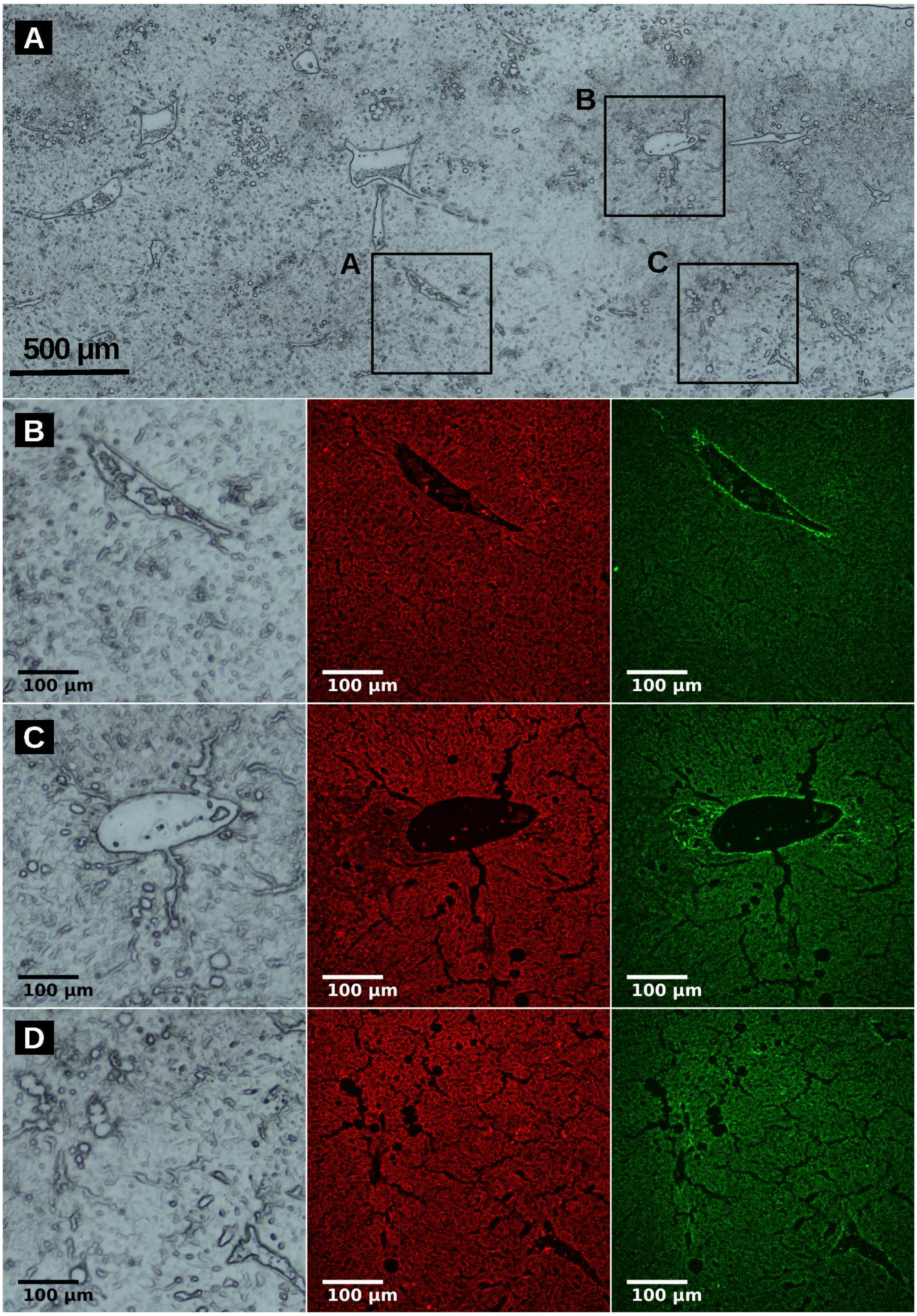

Figure S2

Control: the regions indicated by the red squares in Figure S1 are presented together with the corresponding SHG and TPEF images.

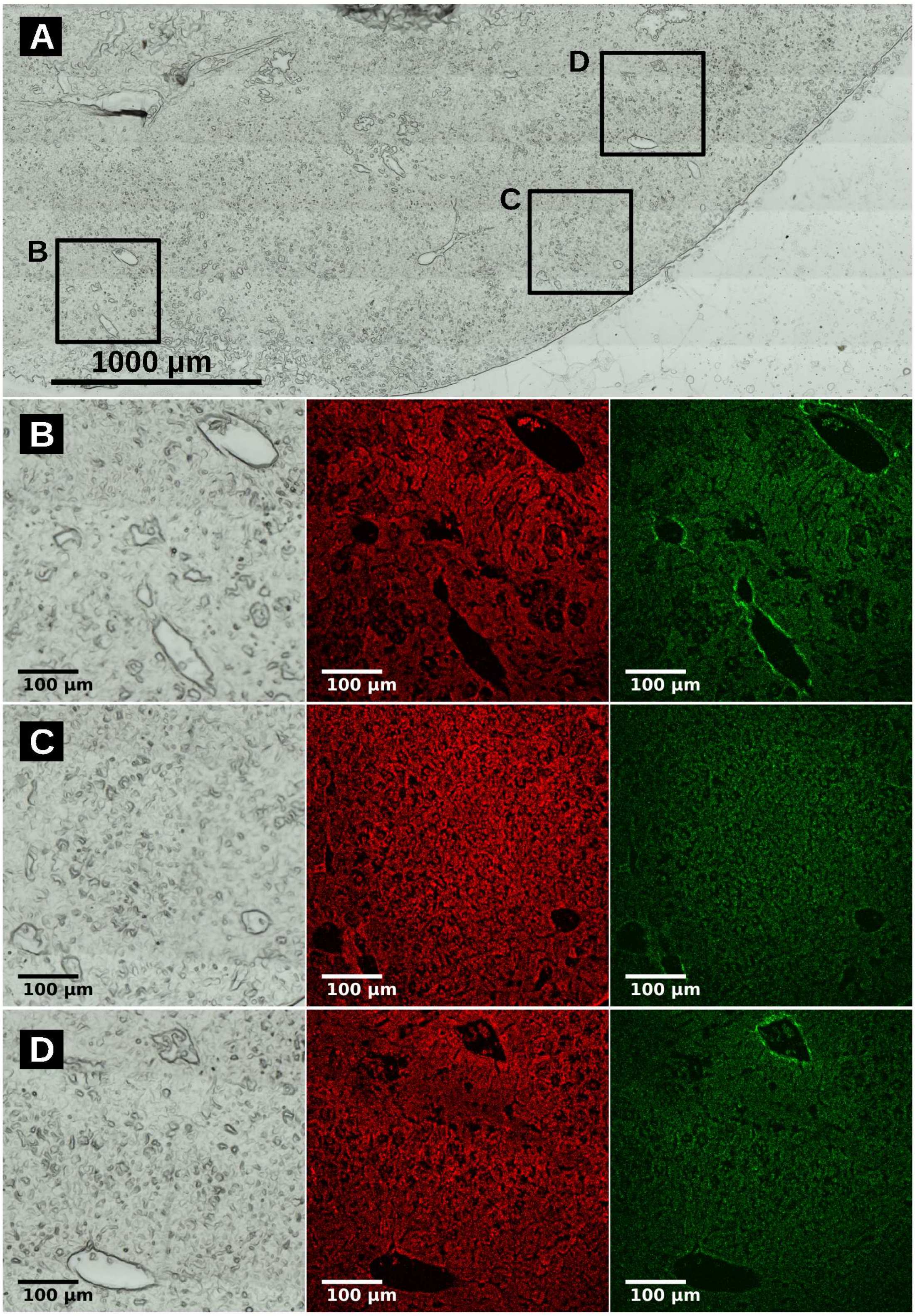

Figure S3
30 days: the regions indicated by the red squares in Figure S1 are presented together with the corresponding SHG and TPEF images.

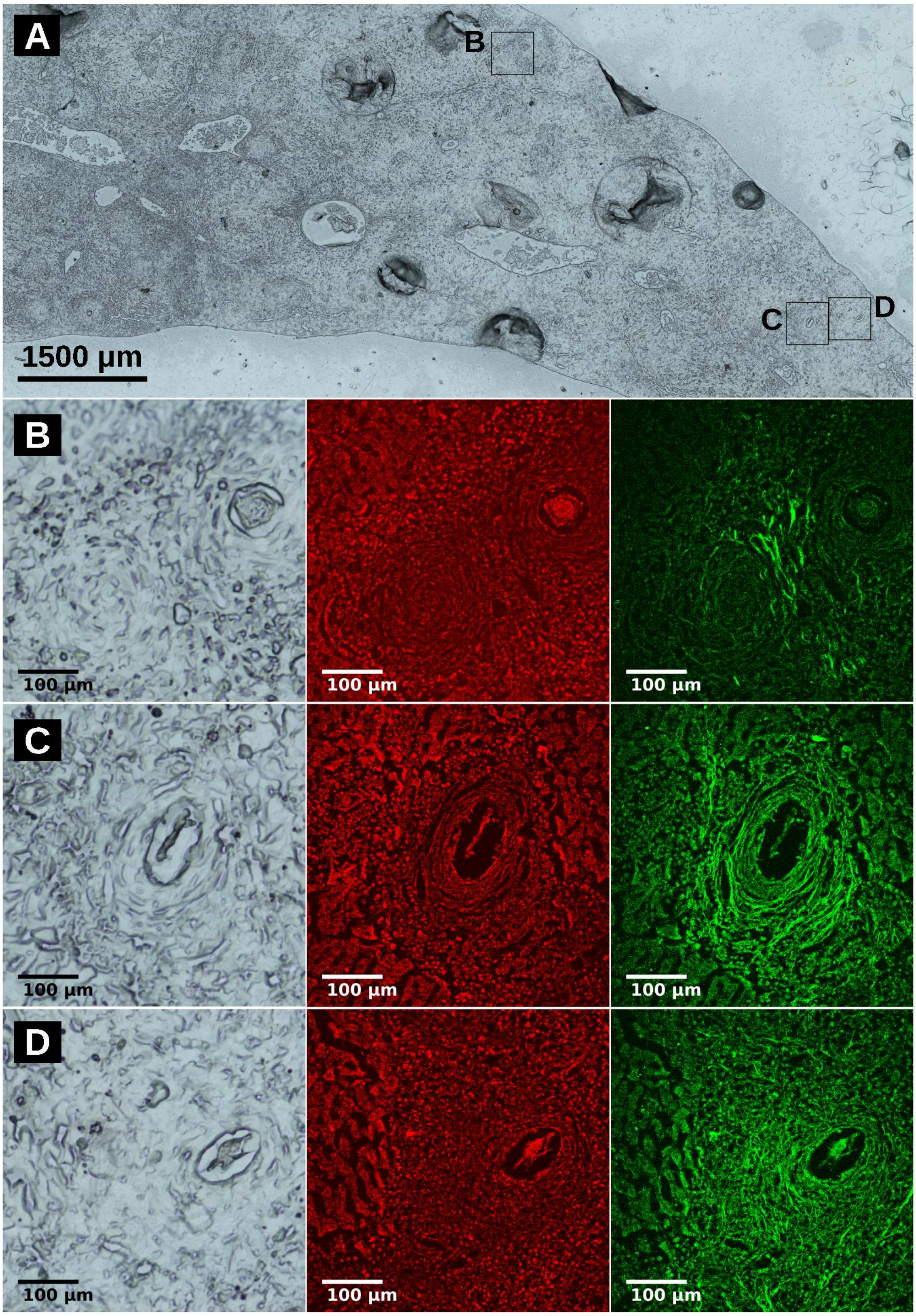

Figure S4
60 days: the regions indicated by the red squares in Figure S1 are presented together with the corresponding SHG and TPEF images.

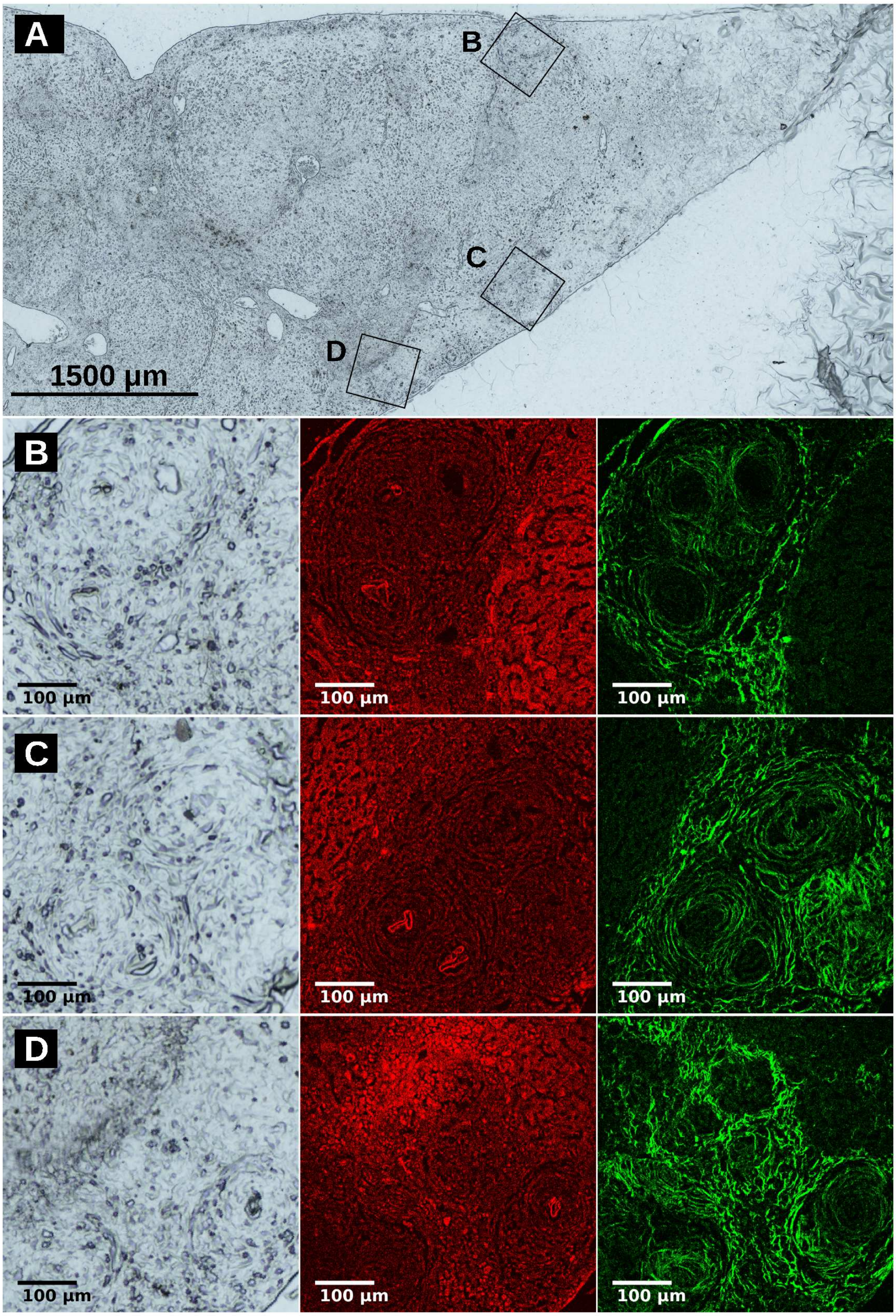

Figure S5
120 days: the regions indicated by the red squares in Figure S1 are presented together with the corresponding SHG and TPEF images.

Random Forest parameter importance

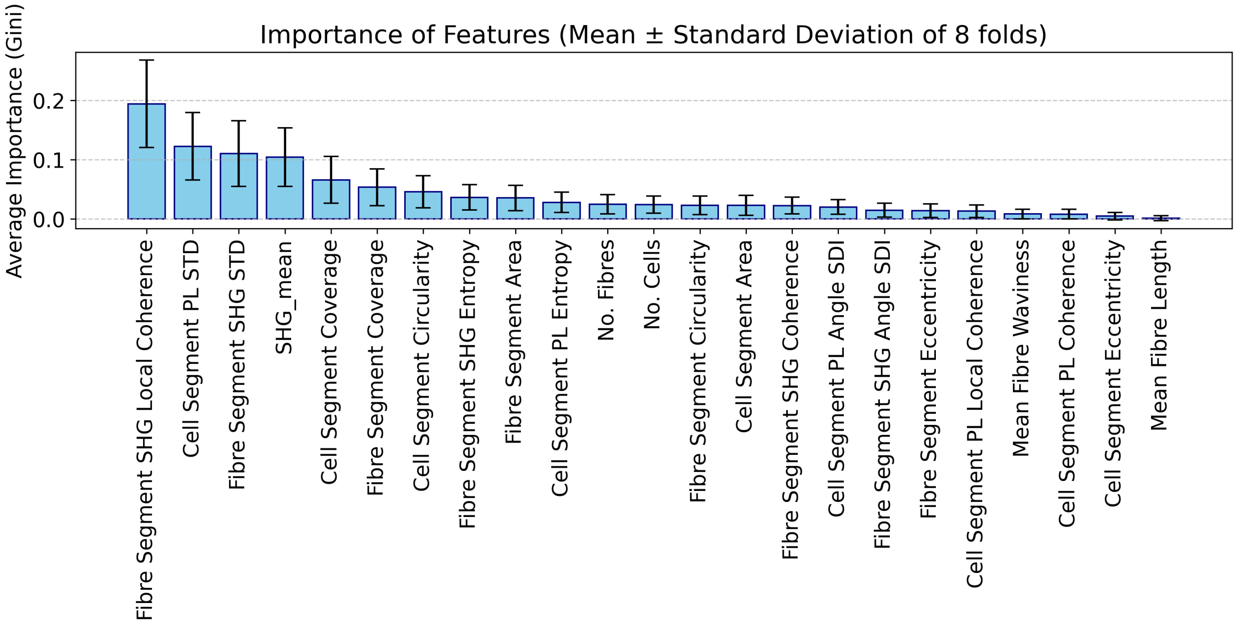

Figure S6
Mean relative importance of the selected features by the Random Forest classifier algorithm.

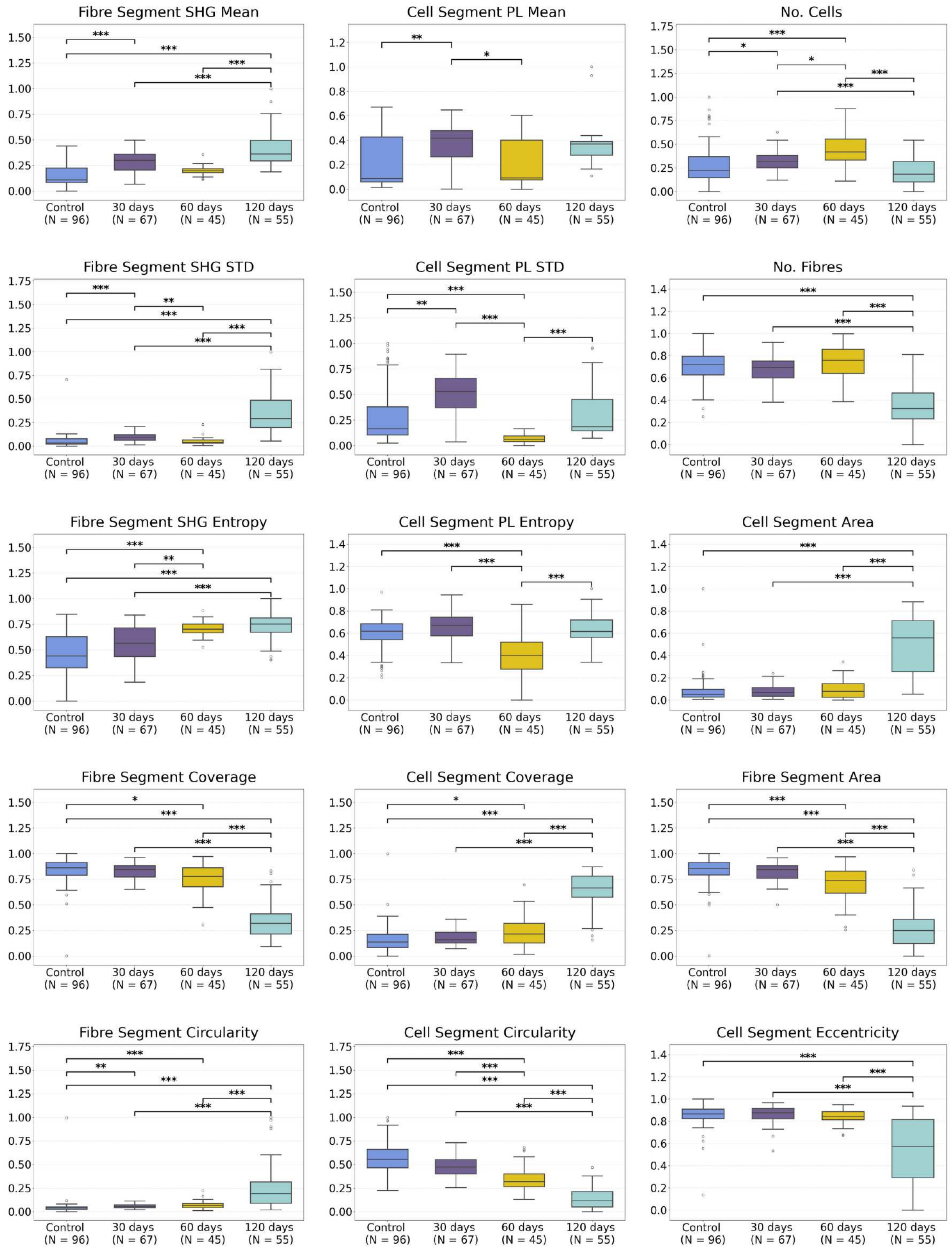

Figure S7
Boxplots for the parameters extracted by the PyFibre software for all the images in each group. Significance between the groups is indicated by: * for $p < 0.05$, ** for $p < 0.01$, and *** for $p < 0.001$.

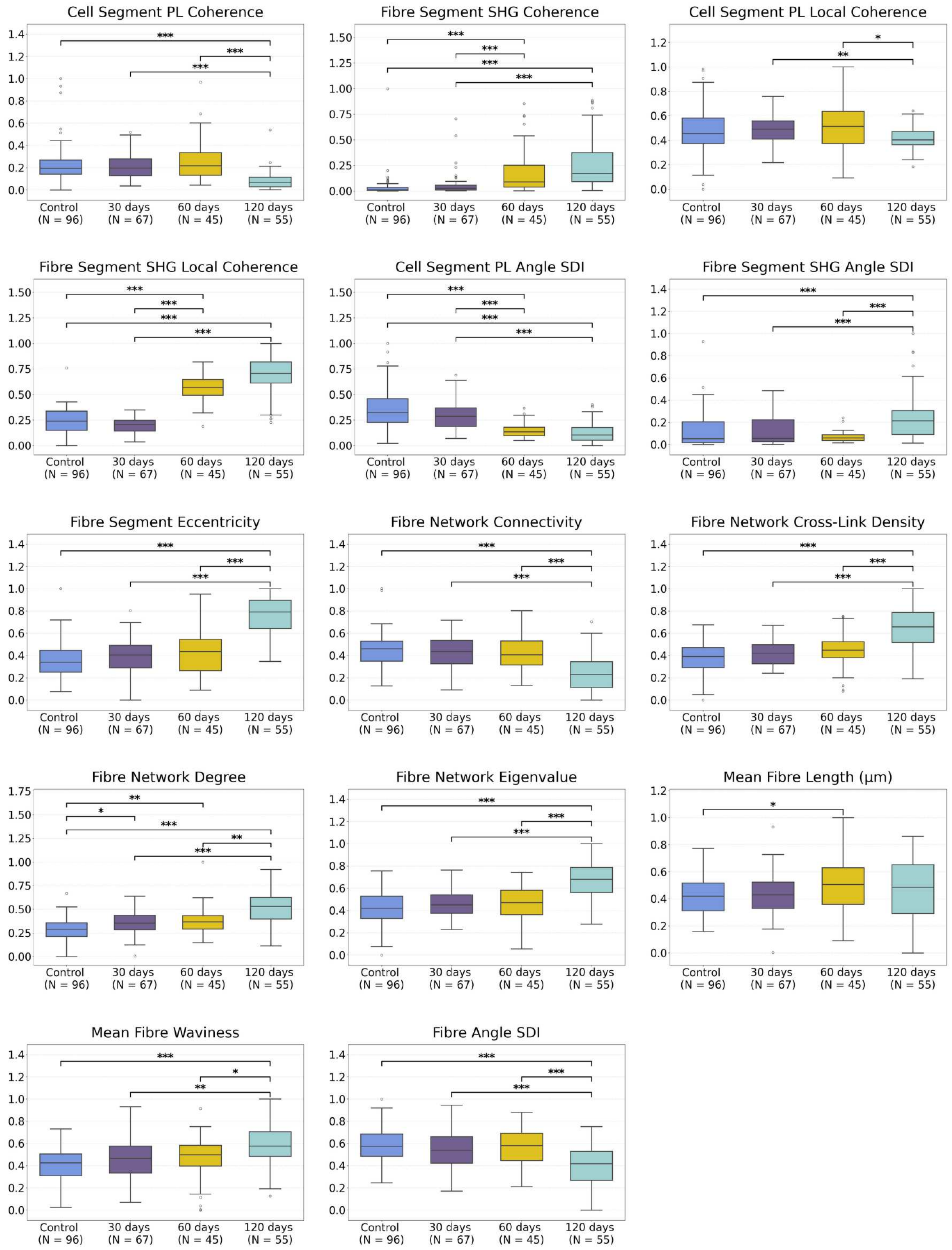

Figure S8

Boxplots for the parameters extracted by the PyFibre software for all the images in each group. Significance between the groups is indicated by: * for $p < 0.05$, ** for $p < 0.01$, and *** for $p < 0.001$.
